# Multiplatform genome-wide identification and modeling of functional human estrogen receptor binding sites

**DOI:** 10.1186/gb-2006-7-9-r82

**Published:** 2006-09-09

**Authors:** Vinsensius B Vega, Chin-Yo Lin, Koon Siew Lai, Say Li Kong, Min Xie, Xiaodi Su, Huey Fang Teh, Jane S Thomsen, Ai Li Yeo, Wing Kin Sung, Guillaume Bourque, Edison T Liu

**Affiliations:** 1 Estrogen Receptor Biology Program, Genome Institute of Singapore, 60 Biopolis Street, Republic of Singapore 138672; 2 Information and Mathematical Sciences Group, Genome Institute of Singapore, 60 Biopolis Street, Republic of Singapore 138672; 3 Microarray and Expression Genomics Laboratory, Genome Institute of Singapore, 60 Biopolis Street, Republic of Singapore 138672; 4 Department of Microbiology and Molecular Biology, Brigham Young University, 753 WIDB, Provo, UT 84602, USA; 5 Institute of Materials Research and Engineering, 3, Research Link, Republic of Singapore 117602

## Abstract

**Background:**

Transcription factor binding sites (TFBS) impart specificity to cellular transcriptional responses and have largely been defined by consensus motifs derived from a handful of validated sites. The low specificity of the computational predictions of TFBSs has been attributed to ubiquity of the motifs and the relaxed sequence requirements for binding. We posited that the inadequacy is due to limited input of empirically verified sites, and demonstrated a multiplatform approach to constructing a robust model.

**Results:**

Using the TFBS for the estrogen receptor (ER)α (estrogen response element [ERE]) as a model system, we extracted EREs from multiple molecular and genomic platforms whose binding to ERα has been experimentally confirmed or rejected. *In silico *analyses revealed significant sequence information flanking the standard binding consensus, discriminating ERE-like sequences that bind ERα from those that are nonbinders. We extended the ERE consensus by three bases, bearing a terminal G at the third position 3' and an initiator C at the third position 5', which were further validated using surface plasmon resonance spectroscopy. Our functional human ERE prediction algorithm (h-ERE) outperformed existing predictive algorithms and produced fewer than 5% false negatives upon experimental validation.

**Conclusion:**

Building upon a larger experimentally validated ERE set, the h-ERE algorithm is able to demarcate better the universe of ERE-like sequences that are potential ER binders. Only 14% of the predicted optimal binding sites were utilized under the experimental conditions employed, pointing to other selective criteria not related to EREs. Other factors, in addition to primary nucleotide sequence, will ultimately determine binding site selection.

## Abstract 2

Refinement of the functional human estrogen receptor binding site model using a multi-platform genome-wide approach reveals extended binding specificity signal.

## Background

Estrogen receptors (ERs) are members of the nuclear receptor superfamily of transcription factors, which plays key roles in human development, physiology, and endocrine-related diseases [1]. Two ER subtypes, namely ERα (*ESR1*) and ERβ (*ESR2*), mediate cellular responses to hormone exposure in target tissues, and receptors are directed at *cis*-regulatory sites of target genes via interactions between the zinc finger motifs in their DNA-binding domains and specific nucleotide sequence motifs termed estrogen response elements (EREs). Specificity protein (Sp)-1 and activator protein (AP)-1 transcription factors are also known to tether with ER and regulate a smaller subset of target genes through Sp1 and AP1 binding sites. The importance of these sites to the overall ER biologic response remains unclear.

The consensus ERE sequence (5'-GGTCAnnnTGACC-3') was derived from conserved regulatory elements found in *Xenopus *and chicken vitellogenin genes and consists of palindromic repeats separated by a three-base spacer to accommodate interactions with receptor dimers [2,3]. Subsequent characterizations of EREs in additional target genes, however, indicate that the majority of response elements deviate from the described consensus sequence [4]. Furthermore, ERE-like sequences are ubiquitous in the human genome, and evidence for ER binding among the majority of ERE-like sites in estrogen response gene expression studies is apparently absent; these factors suggest that additional sequence motifs and/or chromatin features may contribute to the specificity of ER binding and transcriptional response. Recent efforts to model better the ERE by using position weight matrices (PWMs [5]) in order to describe all previously published EREs have resulted in more complete models but with a limited ability to predict *bona fide *ER binding [6,7]. We posited that the current major challenge with construction of ERE models is the limited datasets available, both for experimentally determined ER-bound sites and for ERE-like sites that do not bind ER.

**Figure 1:**
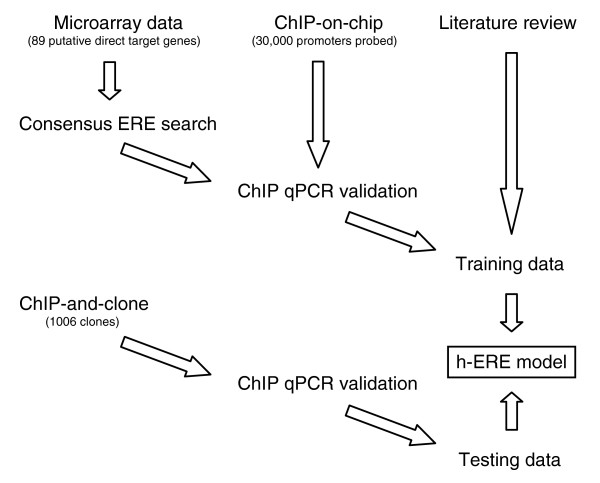
Schematics of ERE discovery and validation for model training and testing. ERE, estrogen response element; ChIP, chromatin immunoprecipitation; qPCR, quantitative polymerase chain reaction.

In addition to compiling the known sites reported in the literature, we pursued a combined experimental and informatics approach to identify additional ER binding sites and their associated direct target genes. This information was analyzed to develop a more faithful model of the ER binding site motifs. To accomplish this, we applied three experimental strategies for ER-binding sites discovery. First, we predicted putative EREs in the promoter regions of direct target genes discovered by microarray analysis [8] and then tested for ER binding at predicted sites of responsive genes by chromatin immunoprecipitation (ChIP) assays [9]. Second, we surveyed ER-binding sites in promoter regions of the human genome by hybridizing fluorescently-labeled ChIP DNA fragments to high-density oligonucleotide arrays ('ChIP-on-chip') with probes against about 30,000 proximal promoters (-1 kilobase [kb] to +0.2 kb relative to the transcription start sites [TSSs]). Third, we detected ER-binding sites across the genome by ChIP, followed by cloning and sequencing of bound fragments ('ChIP-and-clone'). ERE-like sites that have been validated, for binding and nonbinding, by conventional ChIP followed by quantitative polymerase chain reaction (qPCR) using site-specific primers were then used to train and test a model for functional EREs (summarized in Figure 1). In the present study, we focused on functional human EREs to minimize potential noise introduced by species-specific variation, which we have previously observed [8].

## Results

### Functional estrogen receptor binding sites

We used a combination of literature search and direct experimentation to generate a list of qualified ER-binding sites. In this study we constrained ourselves to using only sites that have been validated for the modeling of functional EREs. We first extracted human ERE sequences that have been experimentally validated in the literature to either bind or not to bind ER. Klinge [4] and Bourdeau and coworkers [10] each described EREs that have been validated by electrophoretic mobility shift assays, transient transfection with reporter gene constructs, or ChIP assays.

Supplementing the list of confirmed EREs gleaned from the literature, we experimentally identified functional ER-binding sites using two whole-genome experimental strategies. The first strategy was to extract candidate ER-binding sites computationally from a list of putative direct ER target genes. Eighty-nine putative direct target genes were identified as genes expressed in MCF-7 cells that were responsive to estradiol treatment, sensitive to inhibition by Faslodex (ICI 182,780), and insensitive to cycloheximide [8]. We then computationally surveyed 3.5 kb regions flanking the TSSs (-3 kb to +0.5 kb) of these 89 genes to identify proximate consensus EREs (allowing for deviations in up to two conserved positions of the consensus motif). Each site was then tested by ChIP assays and qPCR with site-specific primers to determine the true nature of ER binding. Eight EREs were found to be bound by ER, whereas 41 others were not found to be bound by ER.

In our second approach, we performed ChIP assays on estradiol-treated breast tumor cells and detected ER-binding sites using high-density oligonucleotide microarrays (NimbleGen, Madison, WI, USA) containing probes against proximal promoter regions (-1 kb to +0.2 kb from TSS; 12 probes per promoter) of over 30,000 human known gene and RefSeq transcripts annotated in the human genome sequence hg16 (July 2003), NCBI build 34 annotation of the UCSC genome browser. The ChIP-on-chip studies were performed using duplicate array experiments on the ChIP samples and on input control DNA. The promoters that appeared among the top 5% of the binding ratio range (ER antibody versus control) for both replicates, that had at least a 15% increase, and that were supported by consistent binding ratio enrichment across more than four probes or additional evidence of ER regulation from the microarray data were selected. Putative EREs (allowing for up to two mismatches from the consensus) were then identified in the selected promoters, and some were further validated by additional ChIP and qPCR (see Materials and methods, below, for more detail). Out of the total 28 sites tested, 13 were found to bind ER whereas 15 were not. From the literature sources and experiments described above, a total of 45 validated ER-binding sites and 58 validated non-ER-binding were identified, all of which bore close resemblance to the consensus ERE (Table 1). Each of the 45 binders and 58 non-binders was associated with a gene and most were located in the genes' upstream regulatory regions. This list of 103 genes were used as the training set to assess the significance of ancillary sequence signals beyond the core ERE that might better predict ER binding.

### Ancillary signals for ER binding around the core ERE

ER is known to interact with the 10 base pair (bp) long consensus ERE (hereafter referred to as the 'core ERE'). Presence of the consensus site (or its acceptable variants) is required for the direct binding of the ER dimer to the DNA. However, it is still unclear whether the core site alone is sufficient to signal activated ER for such binding or whether additional ER-binding signals in the sequences flanking the core can be used to distinguish binders from nonbinders. An *in silico *supervised learning experiment was devised to explore these possibilities.

We modeled the problem of finding additional signals for ER binding among the sequences surrounding the core ERE as a binary classification problem (binders versus nonbinders). The features were position-specific motifs surrounding the core ERE. In other words, we asked whether there is any motif (*m*) within a definitive distance (*p*) to the core ERE that could help distinguish the binders from nonbinders. The robust and versatile naïve Bayesian classification approach was employed, with binary tuple <*m*,*p*> as features, where *m *is a *k*-bp long motif and *p *is the distance between motif *m *and the core ERE. Two sets of experiments were set up. The first consisted of the core plus its flanking regions, whereas the second considered only the flanking regions of core ERE. The motif length *k *and the size of flanking regions were similarly varied in both setups. The goal was to learn whether motifs of certain length at particular distances from the core could contribute to the discrimination of binders from nonbinders. Although the results indicated that window size (*k*) of 1 bp generally outperformed the rest (Additional data file 1), the span of flanking regions did not appear to affect significantly the outcome of the two experiments.

**Figure 2:**
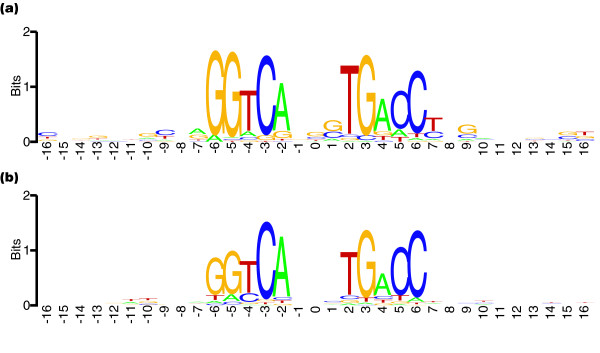
Sequence logos. Shown are sequence logos for **(a) **the 45 ER-binding loci with 10 bp flanking sequences and **(b) **58 ER nonbinding loci with 10 bp flanking sequences. The logo for the binders exhibited additional signal at the third bases upstream and downstream of the core palindromic ERE. bp, base pairs; ER, estrogen receptor; ERE, estrogen response element.

These observations suggested that additional signal for ER-binding might lie in the distribution of single nucleotides adjacent to the core ERE. This hypothesis was initially investigated by visually inspecting the sequence logo [11] constructed from the binders, including their flanking sequences. Shown in Figures 2a (for ER binders) and 2b (for nonbinders) are the logos for up to 10 flanking nucleotides. Comparison between the binders and nonbinders revealed that additional binding signals potentially came from adjacent nucleotides, specifically those up to 3 bp flanking the core ERE, which extended the consensus palindrome. A series of Monte Carlo runs, performed to estimate the probability that observing such additional signals could happen by chance alone, showed that the signals are statistically significant at 3 bp away from the core motif (Monte Carlo *P *value = 0.002 and *P *value < 0.001; see Materials and methods and Additional data file 3).

**Figure 3:**
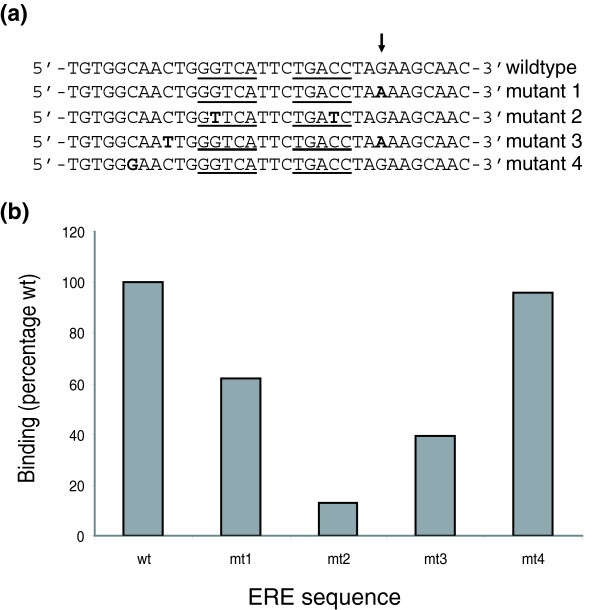
Substitution of the conserved guanine outside of the canonical ERE disrupts ER binding. **(a) **Interactions between ER and wild-type and mutant EREs were measured by SPR. The canonical ERE is underlined, and the conserved guanine is indicated by an arrow. Base substitutions are indicated in bold. **(b) **Binding of ER to ERE is indicated as a percentage of binding relative to the wild-type sequence. ER, estrogen receptor; ERE, estrogen response element; SPR, surface plasmon resonance.

To determine the functionality for the conserved cytosine and guanine three bases upstream of the first ERE half-site and downstream of the second ERE half-site, respectively, we examined the interactions between ER and wild-type and mutant binding sites using surface plasmon resonance (SPR) spectroscopy. Purified ER was incubated with either the previously validated ERE (wild-type) adjacent to the *GREB1 *gene or mutants containing substitutions in the conserved guanine (mutant 1), the canonical half-sites (mutant 2), in the conserved guanine and the cytosine in the symmetrical position upstream of the first ERE half-site (mutant 3; see Figure 3a), and at the sixth bases upstream of the core ERE (mutant 4; see Figure 3a) as the negative control. Substitution of the conserved guanine (mutant 1) disrupted ER binding by about 40%, and, as expected, mutations in the consensus half-sites reduced binding significantly (see Figure 3b). Interestingly, substitution of the cytosine three bases upstream of the first half-site with an adenine (Figure 3b, mutant 3), in addition to the substitution in the conserved guanine adjacent to the second half-site, further diminished binding. As was also expected, the substitution outside the three bases flanking the ERE did not perturb the binding significantly. These results indicate that the conserved guanine outside of the canonical ERE, discovered by modeling novel ER binding site, is involved in mediating ER binding to the ERE.

### Modeling functional EREs

The model we propose, h-ERE, exploits the above observation and consists of two PWMs representing the models for binders and nonbinders. The model relies on a decision tree for classifying sites into binders or nonbinders, based on the scores obtained from the individual PWMs. Two sets of 19 bp sequences, one for binders and the other for nonbinders, were formed from the core sites plus three adjacent nucleotides. We further optimized the binding EREs by minimizing the total entropy of the aligned sites (see Materials and methods), while augmenting the nonbinding EREs by taking both strands of the validated nonbinding loci when constructing the weight matrix.

**Figure 4:**
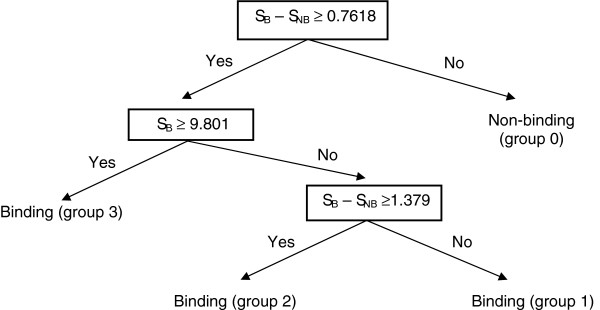
Decision tree for ERE prediction. Group 3 EREs would be predicted to be the highest likelihood binders of ER. ER, estrogen receptor; ERE, estrogen response element; S_B_, binding score; S_NB_, nonbinding score.

With this information we constructed a decision tree for the selection of high-likelihood binding EREs versus nonbinding EREs. Each matrix was used to calculate the log-likelihood of a given 19 bp site to be a binder or a non-binder. For each site two scores can be calculated, the binding score (S_B_) and nonbinding score (S_NB_). Complementing the matrices, a decision tree for distinguishing binders and nonbinders based on S_B _and S_NB _was constructed from all of the training dataset using the CART algorithm [12] implemented in R, with 100 cross-validation runs. Figure 4 depicts the resultant tree. Putative binders are further subcategorized into three groups, from weak binding (group 1) to strong binding (group 3). Apart from these groupings, sites whose raw log-likelihood binding score (S_B_) is greater than its nonbinding (S_NB_) scores are potentially functional sites. Additionally, to reflect the nature of the validated sites, the model considers sequences whose core EREs have more than 4 bp mismatches with the consensus ERE, GGTCAnnnTGACC, to be non-binding.

In all, given a 19 bp sequence, the proposed h-ERE first checks whether the core 13 bp nucleotides contains at most four mismatches to the consensus ERE. Next, based on the computed PWM scores, predictions can be made based on four stringency levels: stringent (considers only sites in group 3 to be binders), medium (predicts sites in group 3 and group 2 to be as binders), relaxed (considers sites of groups 1-3 to be binders), and loose (defines sites whose S_B _> S_NB _as binders).

### Unbiased mapping of EREs

In previously described studies conducted to identify EREs, the analyses have largely focused on the 5' *cis*-regulatory regions of direct target genes. However, ChIP analysis of predicted EREs in the extended promoters of 89 putative direct target genes defined by hormone and inhibitor treatments and microarray expression data [8] indicated ER binding in only 9% of the promoter regions from genes apparently directly regulated by ER. These results suggest that ER may target binding sites outside of the canonical 5' promoter regions. Therefore, to discover additional EREs in an unbiased manner and to generate a dataset for testing model performance, we employed the 'ChIP-and-clone' strategy of cloning precipitated DNA fragments into a bacterial plasmid vector, followed by direct sequencing of the inserts to identify ER binding sites. This approach has the potential to sample any region of the genome, as opposed to PCR-based or microarray-based directed strategies, which target specific sites or functional regions, respectively. Anti-ER ChIP was performed on nuclear lysates from estradiol-treated MCF-7 cells, followed by cloning of precipitated binding sites into the pCR-Blunt (Invitrogen, Carlsbard, CA, USA) vector. From the ChIP library, a total of 1006 clones were successfully sequenced and specifically mapped to the human genome. Based on the presence of ERE-like sequences or supporting microarray expression data for ER regulation of the adjacent transcript, 33 clones were selected for subsequent validation by ChIP and site-specific qPCR. An additional 75 clones were randomly selected from those that have neither EREs nor adjacent transcript expression data for further validation (data not shown). Thus, a total of 108 clones were validated (five contained EREs and are supported by microarray expression data, 23 with only EREs and no supporting expression data, five supported by microarray but no EREs, and 75 with neither EREs nor expression data).

The validation results indicate that ERE-like sequences remain the predominant feature of functional ER-binding sites. In the five clones with EREs and supporting microarray expression data for ER regulation, the validation rate was 100%; for the 23 clones that encode EREs but lack supporting expression data, the validation rate was 57% (13/23). In contrast, clones for which no ERE-like sequences were detected, the validation rates were 40% (2/5) and 9% (7/75), respectively, for those with and without supporting expression data for the adjacent gene. A total of 19 EREs were found in the 18 empirically verified ER-bound clones. Interestingly, the five validated clones that contain EREs and are adjacent to genes that were shown to be hormone regulated map to intronic regions of the target genes. This is consistent with our hypothesis that ER may bind outside of the 5' *cis*-regulatory regions of target genes. Moreover, when we tested ERE-like sequences in the promoter region of one of the target genes, *SIAH2*, we did not detect ER binding, suggesting that the intronic ERE is the functional ER binding site (data not shown) for this particular target gene. From this analysis, all EREs that bind ER and did not bind ER in the validation experiments were then used to test model performance (Table 2).

Currently, three other models have been widely used to predict functional EREs: consensus sequence search (allowing for certain mismatches), TRANSFAC matrices using MATCH [13] search algorithm, and Dragon ERE finder [6]. The performance of these models (under different settings) is compared with h-ERE in Table 3. Although h-ERE was not the most sensitive or the most specific, it offered the best balance between the two criteria. With the interest of having a single performance measure that captures the balance between sensitivity and specificity, harmonic means of the two were computed (see van Rijsbergen [14] and Materials and methods). By this measure, h-ERE offers the best balance in performance, even under different stringency settings.

### Whole-genome predictions of ER-binding sites

In order to assign specific ERE predictions, we constructed a decision tree using binding and nonbinding scores from the PWMs (see Materials and methods). The parameters were selected to minimize error on the classification of the training set. We scanned the human genome (UCSC hg17) using the h-ERE decision tree and detected 38,024 putative sites under the 'stringent' criteria, including 3607 EREs encoded by Alu repeats. To assess further the performance of our predictive algorithm, we randomly selected 60 sites predicted to be ER binders by h-ERE (group 3 sites) and 60 nonbinders (group 0 sites) for further experimental validation by ChIP and qPCR. Of the 120 sites, specific primers for qPCR could be designed for only 64 sites, 44 of which are binders whereas 20 are nonbinders. Fourteen per cent (6/44) of the predicted binding sites were shown to bind ER (more than twofold enrichment over control) whereas no binding was detected in any of the sites classified as nonbinders (0/20), suggesting that the false-negative rate is less than 5%. The low rate of false negatives allows us to demarcate in the human genome the global set of EREs that contain the universe of putative true binding motifs. This suggests that, taking into account the 14% validation rate, there would be 5363 validated ER-binding sites within the global optimized ERE set for the MCF-7 cells, under conditions similar to our experimental setup.

We then considered how much of the predictions could be attributed to random occurrences simply by chance alone. A series of Monte Carlo simulations were carried out to estimate the false positive rate of h-ERE. One thousand nucleotide sequences 1 megabase (Mbp) long were generated randomly, governed by the empirical single nucleotide distribution of the human genome (UCSC hg17), and were run through h-ERE. The numbers of predicted binders divided by 1 Mbp was reported as the h-ERE false discovery rate per base pair. Taking a conservative estimate of the noise and extrapolating it, for the human genome (about 3 gigabases [Gbp]) about 33,000 (approximately 86%) were estimated to be false positives, and hence approximately 5000 ER-binding sites are present in the human genome.

Taken together, the convergence of these two analyses suggest that binding site motifs will be subject to statistical noise from random motif generation, but that a consistent number of *bona fide *binding sites, for the MCF-7 cells and under similar conditions as our experimentations, is likely to exist (about 5000).

## Discussion

In this report we describe a combinatorial experimental approach for transcription factor binding site discovery and demonstrate superior performance of the resultant computational model. The experimental strategies presented here address the major problem in binding site modeling, namely the small size of experimental datasets for model training and testing. The unique use of validated nonbinding EREs and examining flanking sequences allowed us to identify a novel feature of the ERE.

Previous efforts to characterize the ERE have included mutagenesis studies and electrophoretic mobility shift assays or DNase footprinting experiments. For example, Driscoll and colleagues [15,16] demonstrated that single mutations in the core ERE can greatly disrupt ER binding. Furthermore, they found that changes in the flanking sequences can also either enhance or disrupt binding, depending on corresponding changes in the core ERE. Their experiments examined up to two bases flanking the core ERE, and they found that an A or T in the position immediately flanking the core ERE is important for optimal ER binding. Their observation is supported by the model we present here (Figure 2). In our study we found additional single nucleotide features flanking the consensus ERE that are associated with binding site functionality. In particular, there is a prevalence of guanines in the third position downstream (or equivalently cytosines in the third position upstream) of the core ERE motif in binders but not in the nonbinders. The functional significance of these newly discovered conserved bases were verified by SPR analysis of ER interaction with wild-type and mutant binding sites (Figure 3). These additional features were included in the h-ERE decision tree and probably contributed to improved model performance. Having both the binding and the nonbinding ERE sequences enabled us to assess the sensitivity and specificity of the h-ERE model as compared with the consensus sequence, TRANSFAC database ERE PWM [7], or the previously published Dragon ERE model [6]. Under the four stringency parameters tested, the h-ERE model exhibited the optimal combination of sensitivity and specificity, as measured using the harmonic means of these two factors, with 44-68% improvements over the other models.

A genome-wide scan for putative functional EREs using the h-ERE models yielded more than 38,000 predicted high-probability ER binding sites (group 3), which we have shown should represent the set of all high-likelihood ER-binding EREs. Experimental validation of randomly selected predicted sites indicated that 14% of the sites bound ER under the conditions tested, which agreed with the conservative estimate of an approximate 86% false discovery rate for ERE-like sequences in the human genome. From the two approaches, we project there to be approximately 5000 functional ER-binding sites in the MCF-7 genome. That only one out of seven of the high-likelihood binding EREs are functionally used may be attributed to several possibilities. First is that flanking sequences more distal than where assessed in the present study may contribute to the selection of a functional ERE. For example, the nature of the chromatin around the ERE, the relative location of basal transcriptional complexes, and the density of adjacent binding of other transcription factors are candidate modulators of ER-binding site selection. Second, we only tested for ER binding using one standard condition and in a single breast tumor cell line. It is probably the case that certain tissue-specific and condition-specific binding events are modulated by the presence or absence of ER co-regulators and epigenetic modifications. The MCF-7 cell line is known to have high levels of ER and to over-express of AIB1 (amplified in breast cancer 1), which is a specific co-regulator of ER [17]. Moreover, cancer cell lines have accumulated many genetic rearrangements and point mutations in their passages, which would further confound the results by rendering good binding sites inactive.

In our strategy, the approximately 38,000 high-likelihood ER-binding sites were identified using a training set biased to the 5' *cis*-regulatory regions of genes. However, when we mapped these approximately 38,000 candidate sites to the genome, only 1821 (about 4.78%) resided within 5 kb upstream and 500 bp downstream of the TSS. The majority (about 36.5%) fell inside genes, about 21.4% were within 100 kb upstream of the TSSs, whereas about 21.3% were located up to 100 kb downstream of the 3' terminus. Approximately 20% were mapped to pure intergenic regions. These findings suggest that the standard mode of identifying transcription factor binding by concentrating on immediate *cis*-regulatory elements will be unrewarding. In addition, these data collectively question the assignment of physiologic functionality to an ERE site using only gel shift and transient transfection assays with the extracted element, because these *in vitro *approaches ignore many of the relevant physiologic conditions.

Previously, we found that many functional ERE binding sites around responsive genes are poorly conserved between human and mouse [8]. Moreover, both evolutionarily conserved and nonconserved ERE sites appeared to be equally functional for ER binding in ChIP assays; therefore, there appears to be little advantage in using evolutionary history to identify functional EREs. For this reason, we did not take ERE conservation across species into consideration, as was introduced by Jin and colleagues [18] in their recent report. Instead, we focused on the rules governing functional ER binding in the human genome.

Our observations raise the intriguing possibility that evolution of estrogen response relies on having a large pool of high-quality candidate EREs widely scattered in the genome, some of which are potentially generated by transposable elements (about 9% of high-likelihood EREs were within Alu elements). With mutational drift and under evolutionary pressures, different binding sites around the same genes could be alternatively used and would not have detrimental effects on overall survival. If these alternative binding cassettes prove beneficial to the organism, then these secondary sites will undergo further positive mutations to enhance the ER interaction. Conservation of mechanisms and functions across species may be a reasonable assumption for highly conserved biologic processes. However, in the case of EREs and estrogen functions in development and physiology, phenotypic and experimental analysis suggest species-specific mechanisms and hormone responses, including binding site usage. Therefore, using conservation as a filter for function is likely to introduce a significant number of false-negative findings in ERE predictions. This view is further supported by two recent studies [19,20] that found that many functional transcription factor binding sites are not conserved in evolution but there is no apparent functional divergence of the cognate regulated genes. With the binding site database that we present here, such hypotheses can now be computationally examined with increased confidence.

## Conclusion

The availability of larger experimentally validated binding site sets allows the construction of more robust binding site prediction algorithms. The proposed h-ERE algorithm employed genome-wide binding site data collected from various types of experiments. It outperformed other existing algorithms for predicting ER binding. That only 14% of the predicted optimal binding sites were utilized under the experimental conditions suggests that there are other selective criteria not related to ERE. Overall, although h-ERE is able to demarcate better the universe of ERE-like sequences that are potential ER binders, factors other than primary nucleotide sequence will ultimately determine binding site selection.

## Materials and methods

### Identification of additional functional EREs

To enlarge the set of validated EREs, we employed a two-pronged approach: ChIP-qPCR validation of putative ERE in the promoters of putative direct target genes; and ChIP-qPCR validation of putative ERE found in promoters identified from ChIP-chip experiment (GEO series ID: GSE5405).

For the first approach, we took the 89 putative direct target genes identified earlier in a gene expression microarray study [8], extracted their 3.5 kb extended promoter regions, and scanned the sequences for ERE-like motifs, allowing for up to two-base variation from the consensus ERE. Only those with specific PCR primers flanking the EREs were included in ChIP validations by qPCR. There were 49 EREs from 35 promoters hat met the above criteria. Of these, eight EREs from seven putative direct target genes were validated to bind ER and the remaining 41 EREs did not bind ER under the experimental conditions tested in this study.

In the second experiment, the ChIP-chip experiments, only promoters appearing among the top 5% of both replicate experiment were selected, amounting to 196 promoters (binomial *P *value = 1.42 × e^-33^). We further increased the stringency by requiring at least a 15% increase of the IP (immunoprecipitation) over the input control in two consecutive probes to further filter out potential noise in the system. This resulted in 111 promoters that met the selection criteria. Out of the total 111 promoters, we performed ChIP and qPCR validation on 28 promoters that bore putative EREs and had either microarray data supporting their regulation by ER or had consistent binding across consecutive probes (more than four). Of these, 13 were validated to bind ER and 15 did not bind ER.

Altogether, the study validated 21 EREs to be bound by ER and 56 EREs not to be bound by ER. They are indicated by the words 'this study' or by the citation of reference 8, respectively, in the references (right-most) column of Table 1.

### Chromatin immunoprecipitation assays

MCF7 cells were estrogen deprived for 24 hours and treated with 10 nmol/l of estradiol for 45 min prior to 1% formaldehyde treatment to crosslink the transcription machinery and the chromatin. Immunoprecipitations were carried out overnight with anti-ERα (HC-20) or irrelevant control anti-glutathione S-transferase (GST) antibodies (Santa Cruz Biotechnology, Santa Cruz, CA, USA) and protein A-sepharose beads (Zymed, San Francisco, CA, USA). Washing and extraction protocols were modified from methods described previously [9], and PCR reactions were carried out in an ABI Prism 7900 sequence detection system (Applied Biosystems, Foster City, CA, USA). Forty cycles of PCR were carried out on precipitated DNA and control input DNA. Amplification products were also assayed for specificity by melting curve analysis at the end of each run. Relative quantifications were carried out by building standard curves for each primer set and using genomic DNA, similar to the input, as the template. Enrichment of ER binding was determined by comparing the relative quantities of anti-ER and control anti-GST products. Sites with more than twofold enrichment over control were considered to be bound by ER (or 'binders').

### SPR analysis of ER-ERE binding

Biotinylated ERE strands (5'-end labeling) and the anti-strands were annealed to form DNA duplexes. The DNA duplexes were then immobilized on the SPR disk (gold-coated glass) using biotin-streptavidin-biotin bridge chemistry. Protein was then applied to bind to the immobilized DNA. The end attachment of DNA ensured sufficient strand flexibility. For DNA immobilization, the gold disks were first cleaned in a ultraviolet/ozone chamber for 5 min, followed by immersing in hot piranha solution (a 3:1 mixture of H_2_SO_4 _and H_2_O_2_) for 2 min. After rinsing with de-ionized water and drying using nitrogen, the disks were immersed overnight in a binary biotin-containing thiol mixture (10% biotin-thiol and 90% ethylene glycol-thiol at a net concentration of 1 mmol/l in ethanol). After rinsing with ethanol followed by a drying step using nitrogen, the disks were ready for streptavidin (Sigma, St. Louis, MO, USA) immobilization (0.1 mg/ml in phosphate-buffered saline) and subsequent biotinylated DNA assembly (1 μmol/l in phosphate-buffered saline). ERα (58-708 nmol/l in 40 mmol/l HEPES-KOH binding buffer [pH 7.4], containing 10 mmol/l MgCl_2_, 200 mmol/l KCl, 0.2% Triton X-100, 2 mmol/l DTT) was then applied to bind to the immobilized DNA. After one cycle of protein binding (about 25 min), 0.1% sodium dodecyl sulfate solution was applied to disassociate the protein-DNA complex and to expose the immobilized DNA for new cycles of ER binding.

The SPR measurements were conducted using a double channel, AutoLab ESPR (Eco Chemie, Utrecht, The Netherlands). In a kinetic measurement mode, molecular adsorption on the gold disks was detected as SPR angle shifts (Δθ in mDeg) over time. The measured Δθ was proportionally related to the amount of adsorbed material, with a mass sensitivity of 120 mDeg = 100 ng/cm^2 ^for protein and DNA. The AutoLab SPR equipment was equipped with a two-channel cuvette, with the sensor disk forming the base of the cuvette. Two DNA strands (50 μl) were then immobilized in different channels, allowing protein binding to two different DNA sequences to be monitored in parallel. The measurements were conducted at room temperature and the noise level was 0.2 mDeg. It is worth noting that the SPR experiment were done under varying concentrations of ERα, and the reported relative binding affinities of Figure 3 were averages obtained from the varied concentration of ERα. The same overall relative profiles were observed for the different mutants.

### Probing for auxiliary signals around core ERE

To detect and identify whether additional ER-binding signals were flanking the core ERE site, an *in silico *experiment was devised. We considered whether the surrounding sequences of core ERE sites can be used to distinguish binders from nonbinders. A naïve Bayesian classification [12] approach was employed, with binary tuples <*m*,*p*> as the feature, where *m *is a motif that is *k *bp long and *p *is the location of motif *m *relative to the core. One can imagine constructing, for each sequence *S*, a binary matrix *M*, with *m *as the row index and *p *as the column index, and the value M_m,p _indicates whether motif *m *is present at position *p*. Such a matrix can be built by running a fixed window of size *k *over the sequence *S *and noting down the location of each motif. The class of sequence *S*, whether it is a binder (*B*) or nonbinder (*NB*), can be predicted through the equation *C*(*S*) below. In our set of experiments, *k *was varied from 1 to 5 bp. During the training, the probability distribution was constructed from the raw motif frequency counts with Laplacian smoothing of adding pseudocount *L *to the raw count.

C(S)=arg⁡max⁡θ∈{B,NB}(Pr⁡(S|θ)=∏m,pPr⁡(Mm,p|θ))
 MathType@MTEF@5@5@+=feaafiart1ev1aaatCvAUfeBSjuyZL2yd9gzLbvyNv2Caerbhv2BYDwAHbqedmvETj2BSbqee0evGueE0jxyaibaiKI8=vI8tuQ8FMI8Gi=hEeeu0xXdbba9frFj0=OqFfea0dXdd9vqai=hGuQ8kuc9pgc9s8qqaq=dirpe0xb9q8qiLsFr0=vr0=vr0dc8meaabaqaciGacaGaaeqabaqadeqadaaakeaacaWGdbGaaiikaiaadofacaGGPaGaeyypa0ZaaCbeaeaaciGGHbGaaiOCaiaacEgaciGGTbGaaiyyaiaacIhaaSqaaiabeI7aXjabgIGiolaacUhacaWGcbGaaiilaiaad6eacaWGcbGaaiyFaaqabaGcdaqadiqaaiGaccfacaGGYbGaaiikaiaadofacaGG8bGaeqiUdeNaaiykaiabg2da9maarafabaGaciiuaiaackhacaGGOaGaamytamaaBaaaleaacaWGTbGaaiilaiaadchaaeqaaOGaaiiFaiabeI7aXjaacMcaaSqaaiaad2gacaGGSaGaamiCaaqab0Gaey4dIunaaOGaayjkaiaawMcaaaaa@5CC7@

A cross-validation like supervised classification experiment was performed upon a sequence set, grouped into two or more distinct classes, by randomly splitting the sequences into 4:1 training and test sets, training the classifier using the training set, and reporting the accuracy of the trained classifier over the test set. One hundred runs of such training/testing were carried out and the accuracy was averaged out. The figure in Additional data file 1 summarizes the outcomes under varied parameter settings.

### Assessing the significance of flanking nucleotide positions

Outcomes of the previous experiments indicated that single nucleotides surrounding the core ERE motif might carry additional discriminating power between binding and nonbinding EREs. In particular, complementary distinguishing nucleotides appeared to be present at the third base pairs after the core ERE, under visual analysis using sequence logos (Additional data file 2). We designed and carried out a Monte Carlo experiment to quantify its significance. Employing information entropy, or roughly the degree of randomness, as the statistics for each nucleotide position, we asked whether the surrounding nucleotide positions of arbitrary ERE-like sites (up to two mismatches to the consensus) in the human genome contains more information (less random or lower entropy) than those observed surrounding the 45 binding EREs. The exact formula for information entropy is as follows:

H(s)=−∑x={A,C,G,T}fxlog⁡2(fx)
 MathType@MTEF@5@5@+=feaafiart1ev1aaatCvAUfeBSjuyZL2yd9gzLbvyNv2Caerbhv2BYDwAHbqedmvETj2BSbqee0evGueE0jxyaibaiKI8=vI8tuQ8FMI8Gi=hEeeu0xXdbba9frFj0=OqFfea0dXdd9vqai=hGuQ8kuc9pgc9s8qqaq=dirpe0xb9q8qiLsFr0=vr0=vr0dc8meaabaqaciGacaGaaeqabaqadeqadaaakeaacaWGibGaaiikaiaadohacaGGPaGaeyypa0JaeyOeI0YaaabuaeaacaWGMbWaaSbaaSqaaiaadIhaaeqaaaqaaiaadIhacqGH9aqpcaGG7bGaamyqaiaacYcacaWGdbGaaiilaiaadEeacaGGSaGaamivaiaac2haaeqaniabggHiLdGcciGGSbGaai4BaiaacEgadaWgaaWcbaGaaGOmaaqabaGccaGGOaGaamOzamaaBaaaleaacaWG4baabeaakiaacMcaaaa@4CF3@

Where *s *is the set of nucleotides and *f*_*x *_is the frequency of nucleotide *x *in the set *s*. Let *s*_*n *_be the set of nucleotides found *n *bp from the core ERE motif. For each flanking nucleotide up to 5 bp upstream and downstream, denoted as *n *= '-5' to *n *= '+5', we took 45 random loci flanking the ERE-like sites in the genome and computed its entropy. This was done 1000 times and the fraction of times it was lower than the observed entropy for the corresponding position of the 45 binding EREs was reported as the estimated *P *value (Additional data file 3).

### Optimizing the sequence set for model building

With the assumption that bindings most probably occur only on one of the strands and that nonbindings mean that none of the strands were bound, we opted to optimize the binder and nonbinder PWMs by minimizing the total information entropy of the binders while augmenting the nonbinder sequence set by taking both strands of the validated nonbinding loci. The total information entropy (TE) can be calculated as follows:

TE(F)=−∑i=1N(∑x={A,C,G,T}(fx,ilog⁡2(fx,i)))
 MathType@MTEF@5@5@+=feaafiart1ev1aaatCvAUfeBSjuyZL2yd9gzLbvyNv2Caerbhv2BYDwAHbqedmvETj2BSbqee0evGueE0jxyaibaiKI8=vI8tuQ8FMI8Gi=hEeeu0xXdbba9frFj0=OqFfea0dXdd9vqai=hGuQ8kuc9pgc9s8qqaq=dirpe0xb9q8qiLsFr0=vr0=vr0dc8meaabaqaciGacaGaaeqabaqadeqadaaakeaacaWGubGaamyraiaacIcacaWGgbGaaiykaiabg2da9iabgkHiTmaaqahabaWaaeWaceaadaaeqbqaamaabmGabaGaamOzamaaBaaaleaacaWG4bGaaiilaiaadMgaaeqaaOGaciiBaiaac+gacaGGNbWaaSbaaSqaaiaaikdaaeqaaOGaaiikaiaadAgadaWgaaWcbaGaamiEaiaacYcacaWGPbaabeaakiaacMcaaiaawIcacaGLPaaaaSqaaiaadIhacqGH9aqpcaGG7bGaamyqaiaacYcacaWGdbGaaiilaiaadEeacaGGSaGaamivaiaac2haaeqaniabggHiLdaakiaawIcacaGLPaaaaSqaaiaadMgacqGH9aqpcaaIXaaabaGaamOtaaqdcqGHris5aaaa@59C7@

Where *F *is a 4 × *N *matrix of relative frequency of each nucleotide at each position, which can be derived from the PWM of the aligned sites. The overall entropy of the binders was minimized through selectively reverse complementing some of the binders using a greedy hill-climbing approach, which resulted in 15 binder sequences being reverse complemented.

### A balanced measure based on sensitivity and specificity

The trade off between achieving high sensitivity and high specificity for a prediction system is well appreciated. Here, we propose the use of a single measure to evaluate the balanced performance between specificity and sensitivity. One simple option is to calculate the arithmetic mean of specificity and sensitivity. Arithmetic means, however, might be misleading when rates are being averaged. Inspired by the usage of the F-measure [14] in the field of information retrieval, calculated as the harmonic mean of precision and recall, we opted for using harmonic mean to quantify the balance between sensitivity (*sn*) and specificity (*sp*), which can be easily calculated using the following equation:

G(sp,sn)=2∗sp∗snsn+sp
 MathType@MTEF@5@5@+=feaafiart1ev1aaatCvAUfeBSjuyZL2yd9gzLbvyNv2Caerbhv2BYDwAHbqedmvETj2BSbqee0evGueE0jxyaibaiKI8=vI8tuQ8FMI8Gi=hEeeu0xXdbba9frFj0=OqFfea0dXdd9vqai=hGuQ8kuc9pgc9s8qqaq=dirpe0xb9q8qiLsFr0=vr0=vr0dc8meaabaqaciGacaGaaeqabaqadeqadaaakeaacaWGhbGaaiikaiaadohacaWGWbGaaiilaiaadohacaWGUbGaaiykaiabg2da9maalaaabaGaaGOmaiabgEHiQiaadohacaWGWbGaey4fIOIaam4Caiaad6gaaeaacaWGZbGaamOBaiabgUcaRiaadohacaWGWbaaaaaa@461C@

### Estimating the amount of false positives

A pertinent question any high-throughput *in silico *prediction scheme is the degree of false positivity. The number of falsely predicted binding sites, from among the 38,024 predicted sites in the human genome (about 3 Gbp), we devised a Monte Carlo simulation to estimate the distribution of false-discovery rate. Nucleotide sequences of 1 million base pairs long were generated by drawing random nucleotides from the same nucleotide distribution as the human genome (UCSC hg17). The sequences were then run through the h-ERE. Sites on the random sequences predicted to be binders by the h-ERE represent the false positives. As the single nucleotide based random sequence generation may not faithfully reflect all the inherent properties of the human genome, conservative estimation the false-positive rate was made, by reporting the 99th percentile. The above simulation was iterated 1000 times to approximate the rate of false positives per million base pairs. At the 99th percentile, the false positive rate was 11 false binders per million base pairs. This is roughly 33,000 false positive sites for the approximate 3 Gbp human genome, or about 86% of the total approximately 38,000 predicted binders.

## Additional data files

The following additional data are available with the online version of this paper. Additional data file 1 is a document summarizing the results of preliminary Naïve Bayesian analysis on the validated binding sites and their immediate surrounding sequences. Additional data file 2 is a document showing the sequence logos for the final binder and nonbinder sets. Additional data file 3 is a document tabulating the Monte Carlo *P *values for the information entropy significance of each base pair location immediately flanking the core ERE.

## Supplementary Material

Additional data file 1 The motif width (window size), size of the flanking regions, and the Laplacian pseudocount (L) were varied. Two sets of sequences were used: (**A**) with the core ERE intact, and (**B**) without the core ERE. Overall, single nucleotides appeared to hold certain discriminative power for separating ER binding from ER non-binding sequences. Click here for file

Additional data file 2 Sequence logos for: (**A**) the 45 binders, after entropy optimization, with 3 bp flanking sequences, shown with its reverse complement, and (**B**) 116 non-binder sites, obtained from taking the both strands of 58 non-binding loci. Click here for file

Additional data file 3 Monte Carlo *P *values for the information entropy significance of each base pair location immediately flanking the core ERE. Click here for file

## Figures and Tables

**Table 1:** Genomic coordinates of ERE-like sequences that have been experimentally validated or rejected as ER-binding

Name	Genomic location	Pattern	Validation	Reference
*PDZK1*	chr1:143,215,756-143,215,768	GGTCAccc**A**G**T**CC	Binding	This study
*ADORA1*	chr1:199,790,269-199,790,281	GGT**T**AgggTGACC	Binding	[10] and this study
*ADORA1*	chr1:199,790,414-199,790,426	GGT**GT**cttTGACC	Binding	This study
*AGT*	chr1:227,156,613-227,156,625	GG**G**CAtcgTGACC	Binding	[4]
*GREB1*	chr2:11,603,634-11,603,646	GGTCAaaaTGACC	Binding	[10]
*GREB1*	chr2:11,615,324-11,615,336	GGTCAtcaTGACC	Binding	[10]
*GREB1*	chr2:11,621,861-11,621,873	**A**GTCAgtgT**C**ACC	Binding	This study
*GREB1*	chr2:11,623,258-11,623,270	GGTCAttcTGACC	Binding	[8,10]
*CYP1B1*	chr2:38,214,993-38,215,005	GGTC**G**cgcTG**C**CC	Binding	This study
*CYP1B1*	chr2:38,215,049-38,215,061	GGTCAaag**C**G**G**CC	Binding	This study
*LTF*	chr3:46,481,739-46,481,751	GGTCAagg**C**GA**T**C	Binding	[10]
*AREG*	chr4:75,676,340-75,676,352	GG**A**CAaggTG**T**CC	Binding	This study
*ELOVL2*	chr6:11,154,748-11,154,760	GGTCAtctTGA**TG**	Binding	This study
*VEGF*	chr6:43,844,381-43,844,393	**AA**TCAgacTGAC**T**	Binding	[4]
*LY6E*	chr8:144,170,802-144,170,814	GG**A**CAagaTGACC	Binding	[10]
*PTGES*	chr9:129,597,654-129,597,666	GG**A**CAgccTG**G**CC	Binding	This study
*CASP7*	chr10:115,428,398-115,428,410	GGTCAgggTGA**A**C	Binding	[10]
*CASP7*	chr10:115,428,492-115,428,504	GGTC**G**gggTGA**A**C	Binding	[10]
*CASP7*	chr10:115,428,572-115,428,584	GGTCAgggTGA**A**C	Binding	[10]
*CASP7*	chr10:115,428,612-115,428,624	GGTCAgggTGA**A**C	Binding	[10]
*CASP7*	chr10:115,428,652-115,428,664	GGTCAgggTGA**A**C	Binding	[10]
*CASP7*	chr10:115,428,689-115,428,701	GGTCAgggTGA**A**C	Binding	[10]
*CASP7*	chr10:115,428,743-115,428,755	GGTCAgggTGA**A**C	Binding	[10]
*CTSD*	chr11:1,741,924-1,741,936	GG**C**C**G**ggcTGACC	Binding	[4]
*PGR*	chr11:100,504,595-100,504,607	GGTCAcca**GCT**C**T**	Binding	[4]
*PGR*	chr11:100,505,180-100,505,192	G**CAGG**agcTGACC	Binding	[4]
*SCNN1A*	chr12:6,355,536-6,355,548	GGTCAgccT**C**ACC	Binding	[10]
*GAPDH*	chr12:6,513,208-6,513,220	GG**A**CAtcgTGACC	Binding	[10]
*ESR2*	chr14:63,879,248-63,879,260	GGTCAggcTG**GT**C	Binding	[4]
*FLJ30973*	chr15:55,670,850-55,670,862	GG**G**CAgtgTG**G**CC	Binding	This study
*FLJ30973*	chr15:55,671,545-55,671,557	GGTCAcccTG**CT**C	Binding	This study
*ABCA3*	chr16:2,319,793-2,319,805	GGTCAcggTG**TT**C	Binding	[8]
*IGFBP4*	chr17:35,849,113-35,849,125	GGTCAttgTGAC**A**	Binding	[10]
*TRIM25*	chr17:52,323,321-52,323,333	GGTCAtggTGACC	Binding	[4], [10]
*BCL2*	chr18:59,136,673-59,136,685	GGTC**G**cca**G**GACC	Binding	[4]
*MGC26694*	chr19:19,035,118-19,035,130	G**T**TCAgagTGACC	Binding	This study
*GRAMD1A*	chr19:40,182,519-40,182,531	GG**C**C**T**ggcTGACC	Binding	This study
*ACTN4*	chr19:43,897,093-43,897,105	GGTCActgTGAC**T**	Binding	This study
*GPR77*	chr19:52,532,131-52,532,143	GGTCActcTGAC**A**	Binding	This study
*C3*	chr19:6,671,884-6,671,902	GGT**GG**cccTGACC	Binding	[4]
*NRIP1*	chr21:15,359,833-15,359,845	GGTCAaagTGACC	Binding	[8]
*TFF1*	chr21:42,659,626-42,659,638	GGTC**C**tggTG**T**CC	Binding	This study
*TFF1*	chr21:42,659,906-42,659,918	**A**G**C**CAagaTGACC	Binding	This study
*TFF1*	chr21:42,660,106-42,660,118	GGTCAcggTG**G**CC	Binding	[4]
*CRKL*	chr22:19,595,695-19,595,707	**A**GTCAatcT**A**ACC	Binding	This study
*TSHB*	chr1:115,283,928-115,283,940	GGTCAgctTGAC**A**	Nonbinding	[10]
*TXNIP*	chr1:142,927,222-142,927,234	GGTCAgtg**G**GA**T**C	Nonbinding	This study
*LOR*	chr1:150,045,850-150,045,862	GGTC**C**aaa**G**GACC	Nonbinding	This study
*GREB1*	chr2:11,622,443-11,622,455	**T**G**C**CAccaTGACC	Nonbinding	This study
*GREB1*	chr2:11,625,143-11,625,155	**T**GTCAatcTG**T**CC	Nonbinding	This study
*EN1*	chr2:119,322,563-119,322,575	GGT**T**AcccTGA**A**C	Nonbinding	This study
*UGCGL1*	chr2:128,563,200-128,563,212	**T**GTCAaaaTG**T**CC	Nonbinding	This study
*UGCGL1*	chr2:128,565,292-128,565,304	**T**GTCAcatTGA**G**C	Nonbinding	This study
*PLGLB1*	chr2:87,884,778-87,884,790	GGTCAgtgTG**C**C**A**	Nonbinding	This study
*SIAH2*	chr3:151,966,545-151,966,557	G**C**TCAtagTG**C**CC	Nonbinding	This study
*ATP13A3*	chr3:195,656,453-195,656,465	GGTCAtta**A**TACC	Nonbinding	This study
*CISH*	chr3:50,626,609-50,626,621	GG**C**CAgag**G**GACC	Nonbinding	This study
*LMCD1*	chr3:8,517,591-8,517,603	GG**CCT**gcaTGACC	Nonbinding	This study
*FLJ22269*	chr4:673,249-673,261	GG**G**CAgagTGAC**T**	Nonbinding	This study
*CCNG2*	chr4:78,433,176-78,433,188	GG**A**CAactTGA**T**C	Nonbinding	This study
*STC2*	chr5:172,689,912-172,689,924	GG**G**CAatgTGA**A**C	Nonbinding	This study
*IL6ST*	chr5:55,327,909-55,327,921	GGT**G**AgcaTGA**T**C	Nonbinding	This study
*PLK2*	chr5:57,792,972-57,792,984	GGT**T**Acag**C**GACC	Nonbinding	This study
*OLIG3*	chr6:137,857,308-137,857,320	**C**GTCAtccT**A**ACC	Nonbinding	This study
*FKBPL*	chr6:32,206,228-32,206,240	GG**C**CAgcc**C**GACC	Nonbinding	This study
*FKBPL*	chr6:32,206,311-32,206,323	**C**G**C**CAccaTGACC	Nonbinding	This study
*SERPINE1*	chr7:100,361,980-100,361,992	G**AC**CAgccTGACC	Nonbinding	This study
*SERPINE1*	chr7:100,362,938-100,362,950	GG**A**CAagcTG**C**CC	Nonbinding	This study
*SERPINE1*	chr7:100,363,852-100,363,864	**T**GTCAaga**A**GACC	Nonbinding	This study
*TSPAN13*	chr7:16,566,080-16,566,092	G**A**T**A**AgtcTGACC	Nonbinding	This study
*BLVRA*	chr7:43,570,289-43,570,301	GGTCActcTG**G**C**T**	Nonbinding	This study
*BLVRA*	chr7:43,570,774-43,570,786	**A**GTCAaccT**T**ACC	Nonbinding	This study
*B4GALT1*	chr9:33,157,593-33,157,605	G**C**TCAacg**C**GACC	Nonbinding	This study
*B4GALT1*	chr9:33,158,622-33,158,634	G**A**TCAgaa**G**GACC	Nonbinding	This study
*DNAJC1*	chr10:22,333,030-22,333,042	G**T**TCAactTG**T**CC	Nonbinding	This study
*GAD2*	chr10:26,545,037-26,545,049	GGTC**G**cagTGACC	Nonbinding	[10]
*CXCL12*	chr10:44,202,437-44,202,449	GGTC**C**agcTG**C**CC	Nonbinding	This study
*CXCL12*	chr10:44,203,283-44,203,295	**T**GTCAaaaTG**G**CC	Nonbinding	This study
*PGR*	chr11:100,509,203-100,509,215	**A**GTCAtgtTGAC**A**	Nonbinding	This study
*DGKZ*	chr11:46,321,832-46,321,844	GG**C**CAtgcTG**G**CC	Nonbinding	This study
*CTSW*	chr11:65,403,499-65,403,511	G**AC**CAgccTGACC	Nonbinding	This study
*C14orf131*	chr14:101,872,078-101,872,090	GG**C**CAacaTGAC**A**	Nonbinding	This study
*DLG7*	chr14:54,727,987-54,727,999	GGTC**G**tcc**A**GACC	Nonbinding	This study
*ESR2*	chr14:63,876,354-63,876,366	G**AC**CAgccTGACC	Nonbinding	This study
*THBS1*	chr15:37,657,943-37,657,955	GGTCAatc**CC**ACC	Nonbinding	This study
*FLJ13710*	chr15:69,737,514-69,737,526	**A**GTCAttgT**T**ACC	Nonbinding	This study
*FLJ13710*	chr15:69,738,257-69,738,269	GGTCAatgTG**CG**C	Nonbinding	This study
*FLJ13710*	chr15:69,738,459-69,738,471	G**C**TCActtTG**T**CC	Nonbinding	This study
*SH3GL3*	chr15:82,077,053-82,077,065	G**A**TC**T**tgcTGACC	Nonbinding	This study
*SMAP-1*	chr15:89,278,745-89,278,757	**A**GTCAatcTG**T**CC	Nonbinding	This study
*ABCA3*	chr16:2,321,166-2,321,178	GGTC**T**tttT**T**ACC	Nonbinding	This study
*HCFC1R1*	chr16:3,015,149-3,015,161	G**AC**CAgccTGACC	Nonbinding	This study
*ADCY9*	chr16:4,107,737-4,107,749	GGTCAggcTG**GT**C	Nonbinding	This study
*ADCY9*	chr16:4,108,935-4,108,947	GGT**G**AaaaTG**T**CC	Nonbinding	This study
*CAPNS2*	chr16:54,100,244-54,100,256	GGTC**C**gtc**C**GACC	Nonbinding	This study
*PAFAH1B1*	chr17:2,441,502-2,441,514	**C**G**C**CAtgtTGACC	Nonbinding	This study
*IGFBP4*	chr17:35,851,519-35,851,531	G**A**TCActgT**A**ACC	Nonbinding	This study
*IGFBP4*	chr17:35,853,510-35,853,522	GGTCAtgcTG**C**CC	Nonbinding	This study
*RBBP8*	chr18:18,766,140-18,766,152	GGTCAttcTG**CT**C	Nonbinding	This study
*MKNK2*	chr19:2,382,491-2,382,503	GG**G**CAgagTGA**G**C	Nonbinding	This study
*BBC3*	chr19:52,426,840-52,426,852	**T**GTCAttgTG**T**CC	Nonbinding	This study
*BBC3*	chr19:52,427,249-52,427,261	GGTCAggcTG**GT**C	Nonbinding	This study
*GPBP6*	chrY:169,893-169,905	G**C**TCAcgaTGAC**G**	Nonbinding	This study

Shown in bold and underlined are nucleotides that deviate from the consensus core ERE. ER, estrogen receptor; ERE, estrogen response element.

**Table 2:** Validation results on genomic loci containing ERE-like sequences identified by sequencing random ChIP fragment from an ER ChIP library

**Nearest gene**	**Genomic location**	**Pattern**	**Validation**
*NBPF4*	chr1:108,492,542-108,492,560	ttaGGTCAgctTG**T**CCcag	Binding
*C1orf21*	chr1:181,327,606-181,327,624	ctgGGTCAgcaTGACCttc	Binding
	chr11:64,942,548-64,942,566	ctgGG**G**CAtgcT**C**ACCtca	Binding
*SEC15L2*	chr2:72,713,948-72,713,966	ggaGGTC**T**aggTGACCtcg	Binding
	chr3:132,571,914-132,571,932	aggGGTCAtggTGAC**A**tta	Binding
*SLC6A6*	chr3:14,429,604-14,429,622	ctgGGTCActgTG**T**CCgga	Binding
*SIAH2*	chr3:151,957,126-151,957,144	acaGGTCAccaTGACCtgg	Binding
*SNX24*	chr5:122,216,372-122,216,390	cagGGT**T**AtctT**A**ACCaac	Binding
*PKIB*	chr6:122,985,938-122,985,956	tttGGTCAtgt**GGG**CCtga	Binding
	chr6:23,720,183-23,720,201	tcgGGTCAtgcTG**C**C**T**ggg	Binding
*BTBD9*	chr6:38,337,561-38,337,579	tggGGTCAtggTGAC**T**cct	Binding
*SHB*	chr9:37,943,504-37,943,522	gcaGGT**GG**ggcTG**C**C**T**cca	Binding
*SLC38A1*	chr12:44,881,783-44,881,801	cag**A**GT**G**AactTGACCtga	Binding
*SLC38A1*	chr12:44,881,800-44,881,818	gagGGTCAtcc**CA**ACCcca	Binding
	chr16:2,781,142-2,781,160	ccaGGTC**G**gctTG**C**CCtta	Binding
	chr16:743,678-743,696	atgGGTCActgTGACCcag	Binding
	chr17:46,382,536-46,382,554	cccGG**A**CAcgaTG**T**CCccc	Binding
*TEX14*	chr17:54,072,183-54,072,201	cacGGTCAtggTGACCtga	Binding
	chr20:54,945,262-54,945,280	ggg**A**G**A**CAcccTGACCtaa	Binding
	chr2:222,089,422-222,089,440	cagG**T**TCAaaaTGACGggt	Nonbinding
*STK10*	chr5:171,535,283-171,535,301	tgtGGTC**T**ctgTG**C**CCagg	Nonbinding
*KIAA1191*	chr5:175,712,328-175,712,346	agaGG**C**CAgtcT**AC**CCtcc	Nonbinding
*RASGEF1C*	chr5:179,478,929-179,478,947	gtgGG**C**C**G**gccTG**G**CCtgt	Nonbinding
*SORCS1*	chr10:108,692,194-108,692,212	cac**A**GTCAtgcTGACCcca	Nonbinding
	chr14:38,648,346-38,648,364	attGGTCAgagTGAC**A**gaa	Nonbinding
	chr14:79,636,926-79,636,944	acc**T**G**G**CAcgcTGACCcat	Nonbinding
*LOC57149*	chr16:20,819,825-20,819,843	tggGGTCAcac**A**G**G**CCcgt	Nonbinding
	chr16:25,535,373-25,535,391	ttaG**T**TCAcctT**A**ACCcct	Nonbinding
*CEACAM6*	chr19:46,954,305-46,954,323	cagG**AC**CAggg**A**GACCtga	Nonbinding

Shown in bold and underlined are nucleotides that deviate from the consensus core ERE. ChIP, chromatin immunoprecipitation; ER, estrogen receptor; ERE, estrogen response element.

**Table 3:** Performance comparison of various prediction algorithms for ER binding using the independent dataset shown in Table 2

**Prediction algorithm**	**Sensitivity**	**Specificity**	**Harmonic mean**	**Fisher's exact test *P *value**
Consensus ERE with ≤2 mismatches	94.74%	30%	45.57%	0.104838
Consensus ERE with ≤3 mismatches	94.74%	0%	0.00%	1
Dragon ERE finder v2.0	68.42%	40%	50.49%	0.477589
TFAC 8.1 (min FP)	31.57%	100%	47.99%	0.057117
TFAC 8.1 (min FN)	78.94%	40%	53.10%	0.255439
h-ERE (stringent)	42.10%	90%	57.37%	0.084693
h-ERE (medium)	68.42%	70%	69.20%	0.056272
h-ERE (relaxed)	73.68%	70%	71.79%	0.03043
h-ERE (loose)	84.21%	70%	76.45%	0.006199

h-ERE outperformed the other algorithms. ERE, estrogen response element.
