# Supplementary material for: Multiplatform genome-wide identification and modeling of functional human estrogen receptor binding sites
Source: Genome Biol. 2006 Sep 9;7(9):R82. doi: 10.1186/gb-2006-7-9-r82 (PMC1794554; doi:10.1186/gb-2006-7-9-r82)
Supplement: Additional data file 1 — The motif width (window size), size of the flanking regions, and the Laplacian pseudocount (L) were varied. Two sets of sequences were used: (A) with the core ERE intact, and (B) without the core ERE. Overall, single nucleotides appeared to hold certain discriminative power for separating ER binding from ER non-binding sequences. [file gb-2006-7-9-r82-S1.doc]

**(A)**

**(B)**

**Figure S1.** Accuracy of Naïve Bayesian classification using position specific motifs as features to classify sequences bound and not bound by ER. The motif width (window size), size of the flanking regions, and the Laplacian pseudocount (L) were varied. Two sets of sequences were used: (**A**) with the core ERE intact, and (**B**) without the core ERE. Overall, single nucleotides appeared to hold certain discriminative power for separating ER binding from ER non-binding sequences.
