# Supplementary material for: Multiplatform genome-wide identification and modeling of functional human estrogen receptor binding sites
Source: Genome Biol. 2006 Sep 9;7(9):R82. doi: 10.1186/gb-2006-7-9-r82 (PMC1794554; doi:10.1186/gb-2006-7-9-r82)
Supplement: Additional data file 2 — Sequence logos for: (A) the 45 binders, after entropy optimization, with 3 bp flanking sequences, shown with its reverse complement, and (B) 116 non-binder sites, obtained from taking the both strands of 58 non-binding loci. [file gb-2006-7-9-r82-S2.doc]

**
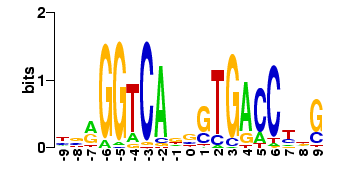

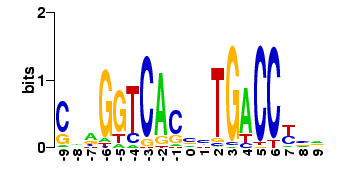
**

**(A)**

**
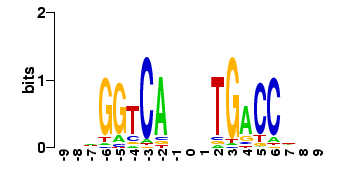
**

**(B)**

**Figure S2**. Sequence logos for: (**A**) the 45 binders, after entropy optimization, with 3bp flanking sequences, shown with its reverse complement, and (**B**) 116 non-binder sites, obtained from taking the both strands of 58 non-binding loci.
