# Supplementary material for: Multiplatform genome-wide identification and modeling of functional human estrogen receptor binding sites
Source: Genome Biol. 2006 Sep 9;7(9):R82. doi: 10.1186/gb-2006-7-9-r82 (PMC1794554; doi:10.1186/gb-2006-7-9-r82)
Supplement: Additional data file 3 — Monte Carlo P values for the information entropy significance of each base pair location immediately flanking the core ERE. [file gb-2006-7-9-r82-S3.doc]

**Table S1**

| **Position** | **-5** | **-4** | **-3** | **-2** | **-1** | **+1** | **+2** | **+3** | **+4** | **+5** |
| --- | --- | --- | --- | --- | --- | --- | --- | --- | --- | --- |
| **Monte Carlo *p*-value** | 0.616 | 0.097 | 0.002 | 0.461 | 0.314 | 0.690 | 0.428 | <0.001 | 0.848 | 0.027 |
